# Circulating *p*-Cresyl Sulfate, Non-Hepatic Alkaline Phosphatase and Risk of Bone Fracture Events in Chronic Kidney Disease-Mineral Bone Disease

**DOI:** 10.3390/toxins13070479

**Published:** 2021-07-10

**Authors:** Jia-Feng Chang, Chih-Yu Hsieh, Jian-Chiun Liou, Kuo-Cheng Lu, Cai-Mei Zheng, Mai-Szu Wu, Shu-Wei Chang, Ting-Ming Wang, Chang-Chin Wu

**Affiliations:** 1 Division of Nephrology, Department of Internal Medicine, En Chu Kong Hospital, New Taipei City 237, Taiwan; cjf6699@gmail.com; 2 Department of Nursing, Yuanpei University of Medical Technology, Hsinchu 300, Taiwan; 3 Division of Nephrology, Department of Internal Medicine, Shuang Ho Hospital, Taipei Medical University, New Taipei City 235, Taiwan; 11044@s.tmu.edu.tw (C.-M.Z.); maiszuwu@gmail.com (M.-S.W.); 4 Research Center of Urology and Kidney, Taipei Medical University, Taipei City 110, Taiwan; 5 Renal Care Joint Foundation, New Taipei City 220, Taiwan; 6 School of Biomedical Engineering, Taipei Medical University, Taipei 110, Taiwan; fish37435@hotmail.com (C.-Y.H.); jcliou@tmu.edu.tw (J.-C.L.); 7 Division of Nephrology, Department of Medicine, Taipei Tzu Chi Hospital, Buddhist Tzu Chi Medical Foundation, New Taipei City 231, Taiwan; Kuochenglu@gmail.com; 8 Division of Nephrology, Department of Internal Medicine, School of Medicine, College of Medicine, Taipei Medical University, Taipei 110, Taiwan; 9 Department of Civil Engineering, National Taiwan University, Taipei 106, Taiwan; changsw@ntu.edu.tw; 10 Department of Orthopaedic Surgery, School of Medicine, National Taiwan University, Taipei 100, Taiwan; dtorth76@yahoo.com.tw; 11 Department of Orthopaedic Surgery, National Taiwan University Hospital, Taipei 100, Taiwan; 12 Department of Biomedical Engineering, Yuanpei University of Medical Technology, Hsinchu 300, Taiwan; 13 Department of Orthopaedic Surgery, En-Chu-Kong Hospital, New Taipei City 237, Taiwan

**Keywords:** *p*-cresyl sulfate, alkaline phosphatase, bone fracture, chronic kidney disease–mineral and bone disorder

## Abstract

Patients with chronic kidney disease (CKD), especially those undergoing hemodialysis, are at a considerably high risk of bone fracture events. Experimental data indicate that uremic toxins intricately involved in bone-related proteins exert multi-faced toxicity on bone cells and tissues, leading to chronic kidney disease–mineral and bone disorder (CKD-MBD). Nonetheless, information regarding the association between *p*-cresyl sulfate (PCS), non-hepatic alkaline phosphatase (NHALP) and skeletal events remains elusive. We aim to explore the association between PCS, NHALP and risk of bone fracture (BF) in patients with hemodialysis. Plasma concentrations of PCS and NHALP were ascertained at study entry. Cox proportional hazard regression analyses were used to determine unadjusted and adjusted hazard ratios (aHRs) of PCS for BF risk. In multivariable analysis, NHALP was associated with incremental risks of BFs [aHR: 1.06 (95% CI: 1.01–1.11)]. The association between the highest PCS tertile and BF risk remained robust [aHR: 2.87 (95% CI: 1.02–8.09)]. With respect to BF events, the interaction between NHALP and PCS was statistically significant (*p* value for the interaction term < 0.05). In addition to mineral dysregulation and hyperparathyroidism in hemodialysis patients, higher circulating levels of PCS and NHALP are intricately associated with incremental risk of BF events, indicating that a joint evaluation is more comprehensive than single marker. In light of the extremely high prevalence of CKD-MBD in the hemodialysis population, PCS may act as a pro-osteoporotic toxin and serve as a potential surrogate marker for skeletal events.

## 1. Introduction

Chronic kidney disease-mineral bone disorder (CKD-MBD) is an intricate systemic complication in CKD patients with abnormal mineral and bone metabolism, typically manifested by dysregulated circulating levels of calcium and phosphorus, intact parathyroid hormone (iPTH), vitamin D, impaired skeletal health and vascular or ectopic soft tissue calcification [1]. Patients with later stages of CKD are doomed to suffer from lower bone mineral density, leading to strikingly higher risk of bone fracture (BF) and subsequent morbidity and mortality [2]. As CKD declines, failing renal clearance results in retention of organic compounds and anorganic substances (e.g., phosphate), also referred to as uremic solutes [3,4]. Among uremic solutes, *p*-cresyl sulfate (PCS) is a protein-bound uremic toxin (PBUT) generated from the metabolism of aromatic amino acids (phenylalanine, tryptophan and tyrosine) by intestinal bacteria [5]. In contrast to phosphate, PCS is difficult to be removed by conventional hemodialysis (HD) modality and thereby accumulates to an extreme degree [5]. Through exerting pro-oxidant and pro-inflammatory effects on complex organ systems, circulating PCS level is an independent predictor of CKD progression and risk of diverse clinical events [6,7,8].

There is growing evidence indicating that both hyperphosphatemia-induced secondary hyperparathyroidism (SHPT) and PBUT accumulation aggravate bone turnover by activating osteoclast activities and increasing the release of calcium and phosphate from bones, leading to bone mineral density (BMD) loss and BF events [4,9,10,11]. Meanwhile, uremic toxins reduce the expression of parathyroid hormone (PTH) receptors and enhance skeletal resistance to PTH in osteoblasts, accounting for a vicious cycle in SHPT [12]. Notably, elevated circulating non-hepatic alkaline phosphatase (NHALP) has been recognized as a robust biomarker for BF events and relevant mortality in high bone turnover diseases [13,14,15]. Nonetheless, the association between PCS, NHALP and BF events remains elusive. Thus, we aim to evaluate the joint effect of PCS and NHALP on BF events in patients with HD.

## 2. Results

### 2.1. Age, Prevalence of DM, HD Vintage, Kt/V Urea, PCS, NHALP, Normalized Protein Catabolic Rate, Albumin, Phosphate, iPTH, Hemoglobin and Hematocrit Levels Were Significantly Different Between BF Event and Event-Free Groups

The baseline bio-clinical data of 352 HD patients with comparisons between BF and BF-free survivors were demonstrated in Table 1. The mean duration of follow-up was 28.4 ± 4.7 months. The overall incidence rate of BFs was 14.5% during 9968 person-months of follow-up, corresponding to an annual event rate of 6.1%. Age, prevalence of diabetes mellitus (DM), HD vintage, Kt/V urea, PCS, NHALP, albumin, normalized protein catabolic rate (nPCR), phosphate, and iPTH levels differed significantly between two groups. The mean age of the whole study group was 64.6 ± 9.3; while the mean age of the group with or without fracture events was 69.9 ± 7.7 and 63.7 ± 9.3 years. Patients with BF had higher prevalence of DM (66.7%) compared to the BF-free group (41.9%) and longer HD vintage (87.4 ± 42.2 vs. 48.6 ± 37.6 months). Notably, the levels of PCS (33.4 ± 21.0 vs. 18.8 ± 12.7 μg/mL), NHALP (117.6 ± 86.8 vs. 81.0 ± 45.6 IU/L), nPCR (1.0 ± 0.2 vs. 1.1 ± 0.2), albumin (3.8 ± 0.5 vs. 3.9 ± 0.4 g/dL), phosphate (6.0 ± 1.5 vs. 5.0 ± 1.4 mg/dL), iPTH (830.3 ± 390.9 vs. 234.3 ± 198.8 pg/mL), hemoglobin (10.2 ± 1.2 vs. 10.8 ± 1.5 g/dL) and hematocrit (31.4 ± 4.1 vs. 31.5 ± 3.8%) were significantly different between the BF event and event-free groups.

### 2.2. BF Event Rates, Kt/V Urea, NHALP, Albumin, Blood Urea Nitrogen, Calcium and iPTH Levels Were Significantly Different among PCS Tertile Groups

Table 2 summarizes the comparison of BF events and relevant bio-clinical data between groups according to circulating levels of PCS categorized as tertiles in the whole study patients. All patients were stratified by the PCS tertiles into three categories: the low, middle and high PCS concentration tertiles were designated as tertile 1 (<16 μg/mL), tertile 2 (16–26.8 μg/mL) and tertile 3 (>26.8 pg/mL). The total BF events in the PCS tertile groups were 10, 15 and 26, corresponding to an annual BF rate of 3.4%, 5.6% and 9.8% respectively. BF events (10, 15, and 26, respectively), Kt/V urea (1.5 ± 0.3, 1.5 ± 0.3 and 1.4 ± 0.3, respectively), albumin (3.8 ± 0.4, 4.0 ± 0.4, and 4.0 ± 0.4, respectively), blood urea nitrogen (57.2 ± 15.2, 59.5 ± 20.5, 62.8 ± 16.0, respectively), calcium (9.3 ± 0.9, 9.2 ± 0.6, and 9.0 ± 0.6, respectively) and iPTH levels (162.7 ± 265.0, 289.9 ± 199.8, and 529.6 ± 349.4, respectively) were significantly different among PCS tertile groups. Notably, levels of NHALP were significantly different and increased in parallel with PCS tertiles (65.6 ± 37.7, 86.5 ± 27.6, and 109.4 ± 78.4 IU/L, respectively). Although the patients in tertile 3 had higher circulating phosphate concentrations and lower hemoglobin levels, the results for statistical difference were insignificant between groups (*p*-value = 0.13 and 0.07, respectively).

### 2.3. The Highest Concentration Tertiles of PCS and NHALP Are Related to an Incremental Risk of BFs

**Figure 1:**
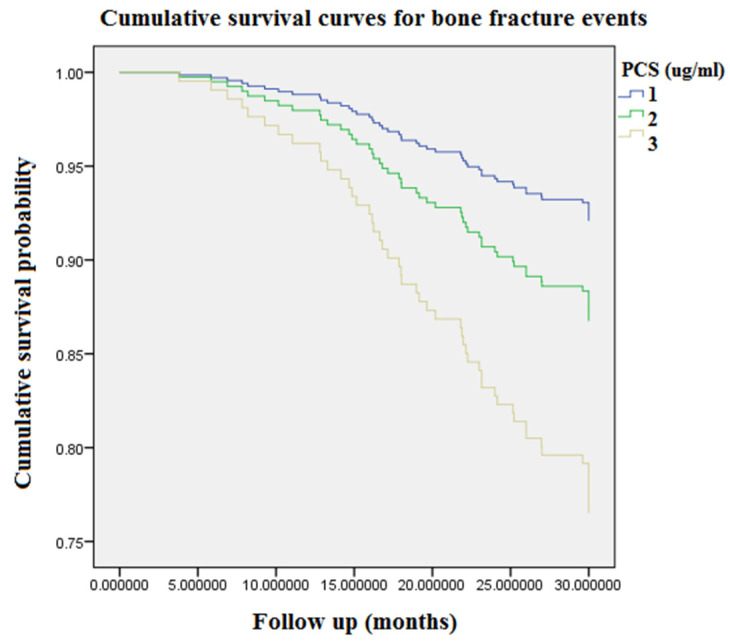
Accumulating event-free survival curves of bone fracture risk among patients with respect to different tertiles of circulating PCS during 9968 person-months of follow-up. Tertile 1, PCS concentration < 16 μg/mL; Tertile 2, PCS concentration = 16–26.8 μg/mL; Tertile 3, PCS concentration > 26.8 μg/mL. PCS = *p*-cresyl sulfate.

**Figure 2:**
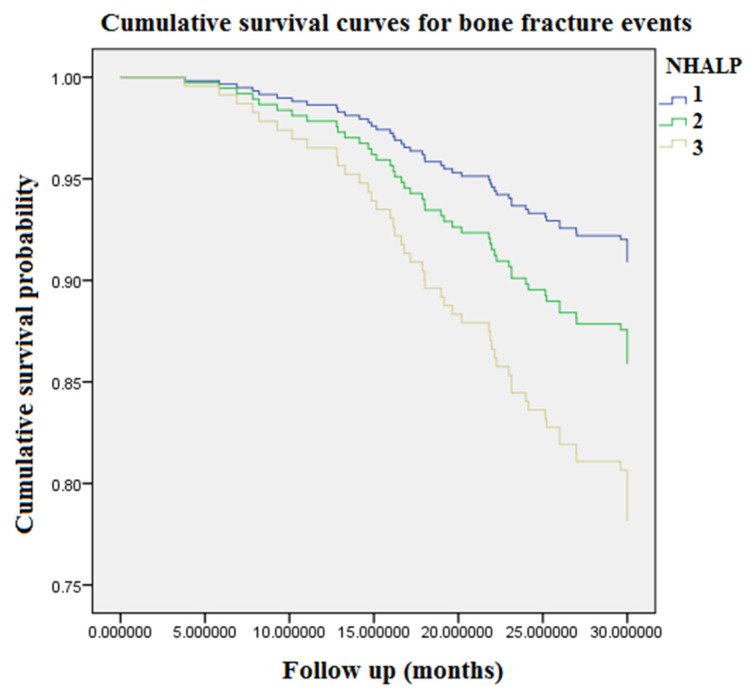
Accumulating event-free survival curves of bone fracture risk among patients with respect to different tertiles of plasma concentrations of NHALP during 9968 person-months of follow-up. Tertile 1, NHALP concentration < 70 IU/L; Tertile 2, NHALP concentration = 70–100 IU/L; Tertile 3, NHALP concentration > 100 IU/L. NHALP = non-hepatic alkaline phosphatase.

**Figure 3:**
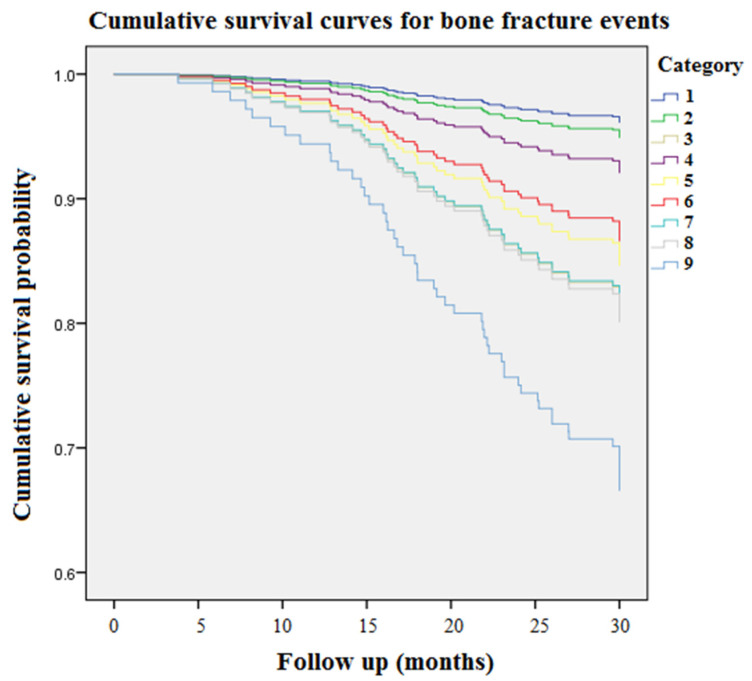
Accumulating event-free survival curves of bone fracture risk among patients with respect to different categories of plasma concentrations of PCS and NHALP during 9968 person-months of follow-up. Category 1, PCS < 16 μg/mL and NHALP < 70 IU/L; Category 2, PCS 16–26.8 μg/mL and NHALP < 70 IU/L; Category 3, PCS > 26.8 μg/mL and NHALP < 70 IU/L; Category 4, PCS < 16 μg/mL and NHALP 70–100 IU/L; Category 5, PCS 16–26.8 μg/mL and NHALP 70–100 IU/L; Category 6, PCS < 16 μg/mL and NHALP concentration > 100 IU/L; Category 7, PCS > 26.8 μg/mL and NHALP 70–100 IU/L; Category 8, PCS 16–26.8 μg/mL and NHALP > 100 IU/L; Category 9, PCS > 26.8 μg/mL and NHALP >100 IU/L. NHALP = non-hepatic alkaline phosphatase. PCS = *p*-cresyl sulfate.

Figure 1 displays accumulating event-free survival curves of BFs among patients with three PCS tertiles during 9968 person-months of follow-up. With respect to tertile 1 (PCS concentration < 16 μg/mL) as a reference group, patients in tertile 3 (PCS concentration > 26.8 μg/mL) were related to an incremental risk of BFs (aHR: 3.25 [95% CI: 1.57–6.73], *p* < 0.05). Figure 2 demonstrated cumulative event-free survival curves of BFs among subjects with respect to different tertiles of plasma concentrations of NHALP: the low tertile 1, NHALP concentration < 70 IU/L; the middle tertile 2, NHALP concentration = 70–100 IU/L; and the high tertile 3, NHALP concentration > 100 IU/L, respectively. With respect to NHALP tertile 1 (<70 IU/L) as reference group, the association between the middle tertile of NHALP (70–100 IU/L) and BF risk was statistically insignificant [aHR: 1.47 (95% CI: 0.71–3.06), *p* value = 0.30]. Notably, the subjects with the high tertile of NHALP (>100 IU/L) were independently associated with an incremental risk of BFs (aHR: 2.3 [95% CI: 1.15–4.52], *p* < 0.05). Figure 3 illustrates cumulative event-free survival curves of BF risk among subjects with respect to different categories of plasma concentrations of PCS and NHALP: Category 1, PCS < 16 μg/mL and NHALP < 70 IU/L; Category 2, PCS 16–26.8 μg/mL and NHALP < 70 IU/L; Category 3, PCS > 26.8 μg/mL and NHALP < 70 IU/L; Category 4, PCS < 16 μg/mL and NHALP 70–100 IU/L; Category 5, PCS 16–26.8 μg/mL and NHALP 70–100 IU/L; Category 6, PCS < 16 μg/mL and NHALP concentration > 100 IU/L; Category 7, PCS > 26.8 μg/mL and NHALP 70–100 IU/L; Category 8, PCS 16–26.8 μg/mL and NHALP > 100 IU/L; Category 9, PCS > 26.8 μg/mL and NHALP >100 IU/L. Patients with a combination of higher PCS tertile (> 26.8 μg/mL) and higher NHALP tertile (>100 IU/L) had the greatest risk of BF events. With respect to the risk of BF events, the interaction term between NHALP and PCS was statistically significant (*p* value for the interaction term < 0.05).

### 2.4. In Multivariate Cox Regression Analysis, the Associations between the Highest PCS Tertile and Risk of BF Events Remain Robust

Table 3 summarizes the independent risk factors of BF events from the unadjusted Cox regression model and PCS tertiles in predicting the occurrence of BF events after multivariate adjustment. Model 1 without adjustment demonstrated that the highest PCS tertile (PCS concentration > 26.8 pg/mL) was independently associated with the risk of BF events (aHR: 3.25 [95% CI: 1.57–6.73]), compared with PCS tertile 1 (<16 μg/mL) as the reference group. Furthermore, age (aHR: 1.05 [95% CI: 1.00–1.10]), DM (aHR: 2.61 [95% CI: 1.46–2.68]), NHALP (aHR: 1.09 [95% CI: 1.05–1.12]), albumin (aHR: 0.53 [95% CI: 0.30–0.95]), HD vintage (aHR: 1.02 [95% CI: 1.01–1.03]), phosphate (aHR: 1.40 [95% CI: 1.19–1.64]), iPTH (aHR: 1.02 [95% CI: 1.02–1.03]) and hemoglobin (aHR: 0.77 [95% CI: 0.63–0.93]) were all independent risk factors of BF events in the current study. Model 2 adjusted for all independent risk factors of BFs demonstrated the highest PCS tertile was associated with BF risk [aHR: 2.87 (95% CI: 1.02–8.09)]. The following independent risk factors of BF events remained strong after multivariate adjustment: age (aHR: 1.06 [95% CI: 1.02–1.11]), NHALP (aHR: 1.06 [95% CI: 1.01–1.11]), HD vintage (aHR: 1.01 [95% CI: 1.00–1.02]), phosphate (aHR: 1.48 [95% CI: 1.26–1.92]), and iPTH (aHR: 1.03 [95% CI: 1.02–1.03]), respectively.

## 3. Discussion

BF events in CKD-MBD stand for the toughest complications that increase the risk of subsequent high rates of hospitalization and mortality dramatically [15,16,17,18]. However, none of the prior research investigated the association between PCS, NHALP and BF events. Therefore, we show a brand-new idea that a joint evaluation of PCS and NHALP for BF events is more comprehensive than a single surrogate marker. Specifically, higher circulating levels of PCS and NHALP interact to enhance the risk of BFs. Several key points in the current study deserve detailed discussion.

Epidemiological studies indicate that, for CKD stages 1 to 2, 3a, 3b, and 4, the incidence of BF rises progressively from 15.0 to 20.5, 24.2, 31.2, and 46.3/1000 person-years, respectively [19]. The BF risk is almost five times elevated in CKD patients with stage 5 disease versus stage 1–2 disease. In accordance with the previous research, our data reveal the overall incidence rate of BFs was 14.5% in the HD population, corresponding to an annual event rate of 6.1% (Table 1). In our cohort study, age, prevalence of DM, HD vintage, Kt/V urea, PCS, NHALP, albumin, nPCR, phosphate, iPTH, hemoglobin and hematocrit levels were significantly different between the BF event and event-free group. Overall, BF patients aged older and had higher prevalence of DM; longer HD vintage; higher levels of PCS, NHALP, phosphate, and iPTH; and lower levels of Kt/V urea, albumin, nPCR, hemoglobin and hematocrit. After stratification into three tertile groups according to circulating levels of PCS (Table 2), the annual BF rates in the PCS tertile groups were increased in parallel with PCS tertiles (3.4%, 5.6% and 9.8% respectively). Moreover, Kt/V urea, NHALP, albumin, blood urea nitrogen, calcium and iPTH levels were significantly different between PCS tertile groups. Accordingly, we examined the associations between PCS tertiles, other clinical risk factors and BF events in a Cox proportional hazard regression model (Table 3). After multivariate Cox regression analysis, the associations between the highest PCS tertile and risk of BF events remained robust. In addition, both the highest concentration tertiles of PCS and NHALP were associated with an incremental risk of BFs (Figure 1 and Figure 2). Most of all, patients with a combination of higher PCS tertile (> 26.8 μg/mL) and higher NHALP tertile (>100 IU/L) show the greatest risk of BF events (Figure 3), suggesting that a joint evaluation is more comprehensive than a sole marker.

In parallel with renal function decline, PBUTs are difficult to remove by HD that accumulate along with persistent iPTH elevation. Mounting evidence indicates that PBUTs aggravate skeletal resistance to PTH though downregulating peripheral PTH receptor expression and inducing inflammation, oxidative stress and apoptosis in osteoblasts [20,21]. Meanwhile, the parathyroid glands have the potential for overriding peripheral PTH resistance, inevitably resulting in high-turnover bone disease in patients with advanced CKD. Such deterioration of bone quantity with prominent bone resorption culminates in extremely high prevalence of BF events in the HD population. Uremic osteoporosis, a new nomenclature of uremic toxin-related bone illness, is a typical manifestation of CKD-induced bone material abnormalities, particularly in bone elasticity [18]. Uremic osteoporosis is a unique concept describing bone illness in CKD-MBD patients, and the pathophysiological background is not fully understood [9]. Furthermore, uremic osteoporosis is similar but somehow different from conventional skeletal osteoporosis in the general population. For example, the decrease of BMD occurrs mainly in hip, but not in spine, and is associated with age and SHPT in CKD patients [22]. Beyond the risk factor of age, the evaluation of BF risk was not simply the same as the general population due to high prevalence of multiple comorbidities and an altered microenvironment for bone in CKD-MBD, e.g., uremic burden, anemia, malnutrition-inflammation complex syndrome, hyperphosphatemia and SHPT, etc. [19,23]. Indeed, conventional risk factors for BFs, such as gender and DM, did not show significant results in univariate and multivariate Cox regression models. In particular, we demonstrated that the assessment of BF risk in CKD patients intricately interacted with circulating PCS and the biomarker of bone metabolism (NHALP). An additional observational study for the mortality associated with BF events, PBUTs or other novel biomarkers is underway to prove that HD patients suffer high frequency of BF as well as subsequent adverse clinical outcomes. Above all, the medical cost for BFs is more than $1 billion annually in the United States [13]. To reduce medical expenditure, the assessment of BF risk and prevention strategies should be considered in nephrology practice.

To monitor and diagnose bone abnormalities, bone biopsy is regarded as the gold standard, but is very invasive and cumbersome. In clinical routine practice, measurements of circulating bone turnover markers are warranted in CKD patients. To investigate it further, we analyzed the baseline bio-clinical data of 352 HD patients with or without BF events and found older age, higher prevalence of DM, higher levels of PCS and NHALP, hypoalbuminemia, anemia, longer HD vintage and hyperphosphatemia and SHPT were associated with greater risk of BFs. NHALP and iPTH were shown to be bone turnover markers that could serve as surrogate tools for BF risk assessment, corresponding to recommendations of the Kidney Disease Improving Global Outcomes (KDIGO) 2017 revised version [24]. Managing risk factors associated with CKD-MBD is crucial for reducing adverse clinical outcomes. Older age, prevalence of DM, and HD vintage were non-modifiable risk factors, whereas hypoalbuminemia, anemia and hyperphosphatemia were modifiable factors that should be corrected. In the general population, age-related osteoporosis increases the risk of BF events. Beside bone loss, different lipid and polar metabolite profiles were found in bone tissues in older individuals with CKD, surprisingly including the accumulation of uremic toxins [25]. Myriads of studies confirm the cardiovascular and skeletal toxicity of uremic toxins since they share common pathophysiological mechanisms in CKD-MBD [26,27,28], but even so, PBUTs are particularly sticky because of their protein-binding abilities and difficulty to be removed by dialysis. Our study provided novel evidence that the accumulation of circulating PCS could provide clinical predictive value regarding BF events in HD patients.

We acknowledged several limitations about this study. To begin with, we could not infer causality from observational data in the cross-sectional cohort. Next, our cohort constituted of a relatively small sample of an Asian population, such that the results should be interpreted with caution. After that, the circulating biochemical values were obtained at a single time point upon study entry, and variability over time might not be reflected. In addition, the data of BMD monitored by dual-energy x-ray absorptiometry or other bone quality measurements were not performed in our patients. Thus, the severity of osteoporosis was not accessible. Last but not least, we lack information on patient frailty and history of glucocorticoid use, which may significantly affect BF risk.

## 4. Methods

### 4.1. Participants in the Cohort

The Research Ethics Review Committees of En Chu Kong Hospital and Taipei Medical University have approved the study (ECKIRB1070102; TMU-IRB-N201705021) in accordance with the ethical standards of the committee and the Helsinki declaration for research in humans. The study methods were described in detail previously [29,30]. The written informed consent was obtained from the participants of this study. Patients undergoing HD treatment for at least 3 months were eligible for inclusion. All patients had to be older than 18 years of age and be receiving thrice-weekly HD; 388 patients were included. Thirty-six patients were excluded from the study because of unwillingness to participate, terminal illness, death prior to BF events, inadequate dialysis, active infections, advanced cancer, active hepatitis, profound malnutrition, incomplete data or loss of follow-up. The study finally included 352 HD patients with complete medical records and follow-up.

### 4.2. Assessment of Outcomes

The incidence of BF events, which was defined as a new diagnosis of BF for any sites that occurred during follow-up, including skull, all vertebrae bones, ribs, shoulder, humerus, forearm, wrist, hip, pelvis, femur, knee, leg, ankle, fingers, toes or other skeletal bones, were defined as primary outcomes. The occurrence of BF events was evaluated by clinical diagnosis (either from inpatient medical records or outpatient chart review) with evidence of BFs in the formal image report (computed tomography or roentgenogram) by radiologists. Vertebral fractures were assessed with Genant’s method as described previously [13]. The outcome information was centrally assessed by trained clinicians, nephrologists and radiologists.

### 4.3. Measurement of Circulating PCS Levels

We obtained a standard sample of PCS from Alsachim (Illkirch-Graffenstaden, France). The measurement of circulating PCS levels were described in detail previously [6]. Samples containing mixtures of PCS were analyzed with the UHPLC through the Accela 1250 autosampler and separated by a Shiseido HPLC CAPCELL PAK C18 MGII column (150 mm x 1.5 mm, 3.0 μm, Tokyo, Japan). The multiple reaction monitoring (MRM) scanning mode was applied for quantification. We used the Xcalibur software (version 2.2, Thermo-Finnigan Inc., San Jose, CA, USA) to acquire the MS spectra and control the mass spectrometer.

### 4.4. Assessment of Covariates

The following bio-demographic and laboratory parameters of each patient were recorded at baseline: age, gender, hypertension, DM, HD vintage, pre-dialysis blood urea nitrogen, normalized protein catabolic rate, creatinine, potassium, calcium, phosphorus, NHALP, alanine aminotransferase, albumin, uric acid, total cholesterol, triglyceride, hemoglobin, hematocrit, and iPTH. Patients with hepatobiliary diseases were excluded in our study after careful chart review. Thus, the origins of elevated ALP concentrations were determined to be non-hepatic, such as CKD-mineral bone diseases or skeletal events [13]. NHALP is one of the most important osteoblast marker proteins for bone mineralization, hydrolysis of mineralization inhibitor pyrophosphate, and UVC associated mortality in CKD patients [31]. HD vintage was defined as the duration of time between the first day of HD treatment and the first day that the patient entered the cohort. Pre-dialysis blood samples were obtained from existing vascular access for further analyses. All laboratory tests were performed by standard procedures with certified methods.

### 4.5. Statistics

We expressed continuous variables as mean ± standard deviation and categorical variables as number (%). The independence of risk factors associated with BF was investigated using univariate Cox regression analysis. In the Cox regression model, unadjusted and multivariable adjusted hazard ratios (aHRs) of BF risk were calculated for plasma PCS tertiles. The cumulative event-free survival probability and proportional hazards were illustrated with graphical methods. The modification effect between higher NHALP and PCS on BF risk was examined using an interaction product term. A *p* value < 0.05 was considered statistically significant. We used the PASW Statistics SPSS version 22.0 (IBM, NY, USA) to analyze all bio-clinical data of MHD patients.

## 5. Conclusions

In addition to mineral dysregulation and SHPT in HD patients, higher circulating levels of PCS and NHALP are intricately associated with incremental risk of BF events, such that a joint evaluation is more comprehensive than a sole marker. In light of the extremely high prevalence of CKD-MBD in hemodialysis population, PCS may act as a pro-osteoporotic toxin and serve as a potential surrogate marker for CKD–MBD.

## Figures and Tables

**Table 1:** Baseline bio-clinical data with comparisons between bone fracture group and fracture-free survivors in maintenance hemodialysis patients.

	Overall Population (n = 352)	BF Events (n = 51)	Event-Free Survivors (n = 301)
**Age (years)**	**64.6 ± 9.3**	**69.9 ± 7.7**	**63.7 ± 9.3**
Male, *n* (%)	181 (51.4)	25 (49.0)	156 (51.8)
**Diabetes mellitus, *n* (%)**	**160 (45.5)**	**34 (66.7)**	**126 (41.9)**
Hypertension, *n* (%)	180 (51.1)	22 (43.1)	158 (52.5)
**Hemodialysis vintage (months)**	**42 (18.9–80.9)**	**75.8 (58.7–129.2)**	**34.2 (17.1–76.0)**
**Kt/V urea**	**1.5 ± 0.3**	**1.4 ± 0.3**	**1.5 ± 0.3**
**PCS (μg/mL)**	**20.9 ± 15.0**	**33.4 ± 21.0**	**18.8 ± 12.7**
**NHALP (IU/L)**	**86.3 ± 54.9**	**117.6 ± 86.8**	**81.0 ± 45.6**
**nPCR**	**1.1 ± 0.2**	**1.0 ± 0.2**	**1.1 ± 0.2**
Aspartate aminotransferase (IU/L)	16.0 ± 6.7	15.6 ± 5.8	16.1 ± 6.9
Alanine aminotransferase (IU/L)	13.0 ± 9.9	15.0 ± 14.3	12.7 ± 8.9
Total cholesterol (mg/dL)	193.4 ± 48.0	202.8 ± 45.2	191.8 ± 48.3
Triglyceride (mg/dL)	206.5 ± 180.5	239.3 ± 181.3	200.9 ± 180.1
Low-density lipoprotein	106.1 ± 38.1	112.3 ± 40.2	105.0 ± 37.7
Blood glucose (mg/dL)	125.8 ± 60.1	134.3 ± 69.8	124.3 ± 58.3
Blood urea nitrogen (mg/dL)	59.7 ± 17.4	61.3 ± 19.2	59.4 ± 17.1
Creatinine (mg/dL)	10.2 ± 1.8	9.9 ± 1.6	10.3 ± 1.9
Uric acid (mg/dL)	7.4 ± 1.3	7.2 ± 0.9	7.5 ± 1.4
Potassium (mmol L^−1^)	4.5 ± 0.9	4.4 ± 0.7	4.6 ± 0.9
**Phosphate (mg/dL)**	**5.2 ± 1.5**	**6.0 ± 1.5**	**5.0 ± 1.4**
**Albumin (g/dL)**	**3.9 ± 0.4**	**3.8 ± 0.5**	**3.9 ± 0.4**
Calcium (mg/dL)	9.2 ± 0.7	9.2 ± 0.8	9.2 ± 0.7
**iPTH (pg/mL)**	**236.5 (118.0–409.0)**	**745.0 (628.0–925.0)**	**201 (105.0–319.5)**
**Hemoglobin (g/dL)**	**10.7 ± 1.4**	**10.2 ± 1.2**	**10.8 ± 1.5**
**Hematocrit (%)**	**33.1 ± 4.0**	**31.4 ± 4.1**	**31.5 ± 3.8**

Continuous variables were expressed as mean ± SD for Gaussian data, or median (25th–75th percentile) for non-Gaussian data. Categorical variables are expressed as n (%). Boldface represents that the values are significantly different between event-free survivors and non-survivors. iPTH = intact parathyroid hormone; Kt/V urea = dialysis dose calculated by Gotch’s method; NHALP = non-hepatic alkaline phosphatase; nPCR = normalized protein catabolic rate; PCS = *p*-cresyl sulfate.

**Table 2:** Comparison of bio-clinical parameters and BF events based on circulating levels of PCS categorized as tertiles in the whole study population.

	Tertile 1 <16 μg/mL	Tertile 2 16–26.8 μg/mL	Tertile 3 >26.8 pg/mL
Patients, *n* (%)	126 (35.8)	114 (32.4)	112 (31.8)
**BF events, *n* (%)**	**10 (7.9)**	**15 (13.2)**	**26 (23.2)**
**Annual BF rates (%)**	**3.4**	**5.6**	**9.8**
Age (years)	64.1 ± 8.9	64.2 ± 9.0	65.6 ± 10.0
Diabetes mellitus, *n* (%)	58 (46.0)	48 (42.1)	54 (48.2)
Hypertension, *n* (%)	60 (47.6)	56 (49.1)	64 (57.1)
Hemodialysis vintage (months)	41.8 (20.2–71.4)	51.7 (16.6–93.3)	37.3 (20.2–89.5)
**PCS (μg/mL)**	**5.6 ± 4.4**	**22.1 ± 2.4**	**37.0 ± 12.5**
**Kt/V urea**	**1.5 ± 0.3**	**1.5 ± 0.3**	**1.4 ± 0.3**
**NHALP**	**65.6 ± 37.7**	**86.5 ± 27.6**	**109.4 ± 78.4**
nPCR (g/kg/day)	1.1 ± 0.2	1.0 ± 0.3	1.1 ± 0.2
Potassium (mmol L^−1^)	4.6 ± 0.8	4.5 ± 0.9	4.5 ± 0.8
**Blood urea nitrogen (mg/dL)**	**57.2 ± 15.2**	**59.5 ± 20.5**	**62.8 ± 16.0**
Creatinine (mg/dL)	10.2 ± 1.8	10.5 ± 1.9	10.3 ± 1.6
Blood glucose (mg/dL)	130.0 ± 62.1	135.8 ± 79.8	135.6 ± 68.4
Uric acid (mg/dL)	7.6 ± 1.4	7.2 ± 1.0	7.5 ± 1.4
**Calcium (mg/dL)**	**9.3 ± 0.9**	**9.2 ± 0.6**	**9.0 ± 0.6**
Phosphate (mg/dL)	5.0 ± 1.5	5.1 ± 1.4	5.4 ± 1.4
**Albumin (g/dL)**	**3.8 ± 0.4**	**4.0 ± 0.4**	**4.0 ± 0.4**
**Intact parathyroid hormone (pg/mL)**	**96.0 (53.2–139.0)**	**224.0 (175.0–293.0)**	**413.5 (341.0–552.0)**
Hemoglobin (g/dL)	10.8 ± 1.8	10.8 ± 1.2	10.4 ± 1.0
Hematocrit (%)	33.5 ± 5.7	33.5 ± 3.8	32.3 ± 3.2

Continuous variables were expressed as mean ± SD for Gaussian data, or median (25th–75th percentile) for non-Gaussian data. Categorical variables are expressed as *n* (%). Boldface represents that the values are significantly different between PCS tertiles. BF = bone fracture. Kt/V urea = dialysis dose calculated by Gotch’s method. nPCR = normalized protein catabolic rate. PCS = *p*-cresyl sulfate.

**Table 3:** The associations between PCS tertiles, other independent risk factors and bone fracture events in a Cox proportional hazard regression model.

	Model 1	Model 2
	HR (95% CI) *p* Value	HR (95% CI) *p* Value
PCS Tertiles Tertile 3 vs. Tertile 1	3.25 (1.57–6.73) < 0.01	2.87 (1.02–8.09) < 0.05
Diabetes mellitus (yes vs. no)	2.61 (1.46–2.68) < 0.01	1.49 (0.76–2.95) 0.25
NHALP (per 10 unit increase)	1.09 (1.05–1.12) < 0.01	1.06 (1.01–1.11) < 0.01
Age (per year increase)	1.05 (1.00–1.10) < 0.05	1.06 (1.02–1.11) < 0.05
Albumin (per unit increase)	0.53 (0.30–0.95) < 0.05	0.81 (0.35–1.87) 0.62
HD vintage (per month increase)	1.02 (1.01–1.03) < 0.01	1.01 (1.00–1.02) < 0.05
Phosphate (per unit increase)	1.40 (1.19–1.64) < 0.01	1.48 (1.26–1.92) < 0.05
iPTH (per 10 unit increase)	1.02 (1.02–1.03) < 0.01	1.03 (1.02–1.03) < 0.01
Hemoglobin (per unit increase)	0.77 (0.63–0.93) < 0.01	0.82 (0.62–1.10) 0.18

CI = confidence interval; HR = hazard ratio. PCS Tertile 1 indicated PCS concentration < 16 μg/mL; Tertile 3 indicated PCS concentration > 26.8 pg/mL. Model 1: independent risk factors from the unadjusted Cox regression model; Model 2: adjusted for all independent risk factors in the model 1 (PCS, diabetes mellitus, NHALP, age, albumin, HD vintage, phosphate, iPTH, hemoglobin). HD = Hemodialysis; iPTH = intact parathyroid hormone; NHALP = non-hepatic alkaline phosphatase; PCS = *p*-cresyl sulfate.

## Data Availability

The data presented in this study are available from the corresponding author, Chang-Chin Wu, upon reasonable request.
